# Type 1 Diabetes and Disordered Eating - the Rising T1DE: Evolution of a New Service Within a Specialist Eating Disorder Service (SEDU)

**DOI:** 10.1192/bjo.2026.11538

**Published:** 2026-06-30

**Authors:** Mike Apio, Wiebke Hollemann, Jana Ondrova Farmer, Kruti Pandit, Aisha Kibirige

**Affiliations:** Priory Hospital Hayes Grove, Bromley, United Kingdom

## Abstract

**Aims:**

Effectively adapting a SEDU in the management of patients with T1DE – challenges, multi-professional working, capacity, and consent in determining outcomes.

**Background:** Individuals with disordered eating and T1DE pose a challenge across services, due to complications that arise from the interplay of this two-in-one condition, which could be life threatening.

The insidious onset of disturbed eating and its progression to disordered eating behaviours has been recodified in newer diagnostic manuals. Patients with type 1 diabetes mellitus (T1D) remain a source of consternation to professionals and families. Type 1 diabetes mellitus and disordered eating (T1DE), previously known as diabulimia, is not uncommon.

The UK APPG on Eating Disorders emphasise the need for adequate resourcing with integration of services. In response, the Royal College of Psychiatry annexe to MEED addresses diagnoses by highlighting key criteria.

**Methods:**

Re-adapting for T1DE involved: training on T1D with diabetic specialists regarding CGM devices, traditional finger-prick testing, insulin pen management, meal planning and insulin dosing, carb-counting (DAFNE); Implementing the *T1DE Behaviour Checklist*, and our *Quick Guide to Diabetes Management in Patients with T1DE*.

Other interventions: Medical - monitoring pre/post-meal BG, serum ketones etc., Psychology – EDE-Q, PHQ-9, GAD-7, fear hierarchy, OT – MOHO-based assessment of function including exposure, Dietetic - carb and calorie counting tools, Nursing care planning with physical monitoring. Family therapy, goal planning with community ED, PC and KCL national T1DE services and application of the MHA. Pre-screening for neurodevelopmental disorders, personality disorders and common mental health conditions.

**Results:**

150 admissions were recorded over 24 months with complexities such as ASD, ADHD, trauma, personality disorder and self-harm being prevalent. We admitted three of four T1DE referrals – one under section 2 of the MHA, rescinded prior to discharge, and the other informal reassessed and detained under section 3, subsequently rescinded five months later. The third remained informal throughout admission. Outcomes include improvement in binge purging or restriction (>6000kcal to approximate 1800kcal daily), HBs-489C by 30-40%, weight stabilisation, and adherence to insulin doses and administration. There were marked improvements in distress tolerance, EDE-Q and anxiety scores. Two patients returned to employment or university. They responded positively to reinstating antidepressants and one to stimulant treatment.

**Conclusion:**

The service adapted, utilising expert support whilst exploring innovative interventions to facilitate treatment of T1DE. Challenges encountered reinforce the need for clinicians to address barriers and improve engagement by applying a reflective MDT approach. Aligning with patients and family goals is crucial for sustained outcomes.

