# Type 1 Diabetes and Disordered Eating - the Rising T1DE: Evolution of a New Service Within a Specialist Eating Disorder Service (SEDU) – ERRATUM

**DOI:** 10.1192/bjo.2026.12075

**Published:** 2026-07-22

**Authors:** Mike Apio, Wiebke Hollemann, Jana Ondrova Farmer, Kruti Pandit, Aisha Kibirige

Type 1 Diabetes and Disordered Eating - the Rising T1DE: Evolution of a New Service Within a Specialist Eating Disorder Service (SEDU)

Dr Mike Apio, Dr Wiebke Hollemann, Mrs Jana Ondrova Farmer, Mrs Kruti Pandit and Ms Aisha Kibirige

The above abstract published with an error in the fourth sentence of the Results section:

Outcomes include improvement in binge purging or restriction (>6000kcal to approximate 1800kcal daily), HBs-489C by 30-40%, weight stabilisation, and adherence to insulin doses and administration.

The correct sentence follows:

Outcomes include improvement in binge purging or restriction (>6000kcal to approximate 1800kcal daily), HBA1C by 30-40%, weight stabilisation, and adherence to insulin doses and administration.

The publisher apologises for the error.

## References

[ref1] Apio M , Hollemann W , Farmer JO , Pandit K , Kibirige A. Type 1 Diabetes and Disordered Eating - the Rising T1DE: Evolution of a New Service Within a Specialist Eating Disorder Service (SEDU). BJPsych Open. 2026;12(S1)S226.10.1192/bjo.2026.1207542482594

